# Comparison of Clinical Characteristics Between Bullous and Non-bullous Rheumatoid Neutrophilic Dermatosis: A Case Report, Literature Review, and Proposed Diagnostic Criteria

**DOI:** 10.7759/cureus.83427

**Published:** 2025-05-03

**Authors:** Masakazu Kakurai, Kazuma Iwamoto, Yoshihiro Moriyama

**Affiliations:** 1 Dermatology, Tsuchiura Kyodo General Hospital, Tsuchiura, JPN

**Keywords:** acute febrile neutrophilic dermatosis, neutrophilic dermatosis, rheumatoid arthritis, rheumatoid neutrophilic dermatosis, sweet’s syndrome

## Abstract

Rheumatoid neutrophilic dermatosis (RND) is associated with rheumatoid arthritis and typically presents as papules, nodules, and/or plaques bilaterally on the extremities. Rarely, vesiculobullous lesions (bullous RND) may occur. We herein present a case of bullous RND diagnosed in our department. A 70-year-old Japanese woman presented with multiple painful, tense bullae, accompanied by pustules, erythematous papules, and erosions on the lower extremities, and a few hemorrhagic bullae were observed on the soles. Her medical history included seropositive rheumatoid arthritis for 14 years, which was successfully treated with oral prednisolone and tacrolimus hydrate, but joint pain and swelling developed one month before her visit to our department. A skin biopsy of the blister on the lower leg revealed an intraepidermal and subepidermal blister, containing numerous neutrophils. Marked neutrophilic infiltration, showing prominent leukocytoclasis, was observed in the dermis without vasculitis. Direct immunofluorescence yielded negative results. Bacterial cultures from the blisters were sterile. Taken together, the diagnosis of bullous RND was made. Despite treatment with oral minocycline for one week, new skin lesions developed. Treatment was switched to dapsone at 75 mg daily, resulting in the improvement of skin lesions and arthralgia within one week. In this report, we describe a case of bullous RND and compare the differences in clinical findings between bullous and non-bullous RND, which have not been previously documented. Additionally, as RND and Sweet’s syndrome share overlapping clinicopathological features, we proposed five diagnostic criteria for RND: (1) a definitive diagnosis of rheumatoid arthritis; (2) high rheumatoid arthritis disease activity; (3) multiple erythematous papules, nodules, plaques, and/or tense vesiculobullous lesions; (4) predominantly neutrophilic dermal infiltrate without leukocytoclastic vasculitis; and (5) microbial sterility.

## Introduction

Neutrophilic dermatosis (ND) is an inflammatory skin disorder with unique clinical features characterized by a sterile, predominantly neutrophilic infiltrate on histology, and includes Sweet’s syndrome (SS), pyoderma gangrenosum (PG), bowel-associated dermatosis-arthritis syndrome, and rheumatoid neutrophilic dermatosis (RND) [1]. RND, first described by Ackerman in 1978, is associated with severe rheumatoid arthritis (RA) [2]. The condition typically presents as asymptomatic multiple erythematous papules, nodules, and/or plaques bilaterally on the extremities, with a sterile dense neutrophilic dermal infiltrate and no vasculitis on histological examination [3]. Rarely, vesiculobullous lesions may also occur [3]. RND presenting with vesiculobullous lesions (bullous RND) was initially reported by Lowe et al. in 1992 [4].

RA is a systemic inflammatory disorder with both articular and extra-articular involvement, occurring mainly after middle age [5,6]. Female patients with RA outnumber male patients by a ratio of approximately 2.5 to 1 [5]. Extra-articular involvement is common, affecting almost every organ system [5]. Among the extra-articular involvements, specific skin manifestations are diverse and include classic rheumatoid nodules, accelerated rheumatoid nodulosis, granulomatous dermatitis, rheumatoid vasculitis (RV), PG, and RND [5].

Therefore, the diagnosis of RND can be challenging owing to the wide range of clinical manifestations and the variety of skin disorders associated with RA. Although the clinical characteristics of RND have been reported [3], the clinical differences between bullous and non-bullous RND remain unclear. In addition, RND is clinicopathologically similar to SS, and the distinction between the two is still debated, with no diagnostic criteria currently available for RND. Therefore, we aimed to identify the differences in the clinical characteristics of bullous and non-bullous RND and to propose a clinical practice guide for RND. Additionally, this literature reports the clinical and histological features of a patient with bullous RND diagnosed in our department.

## Case presentation

**Figure 1:**
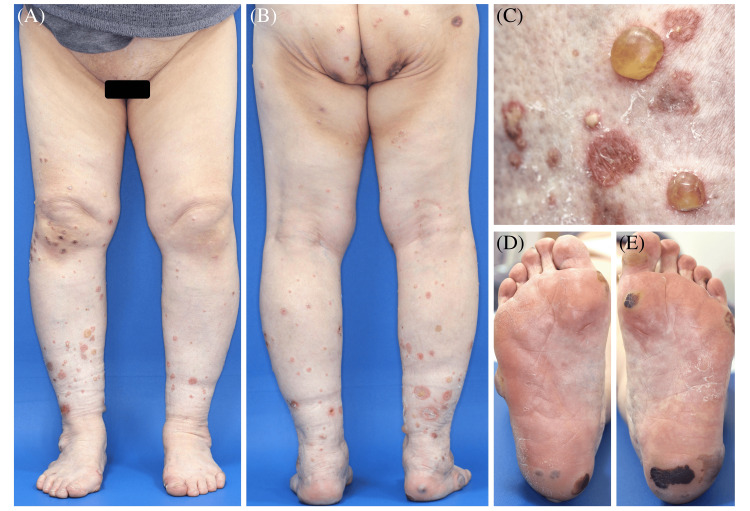
Clinical images of bullous RND experienced in our department (A-C) Multiple painful, tense bullae and blisters, accompanied by pustules, erythematous papules, and erosions on the lower extremities. Mild erythema around the bullae, blisters, and pustules are observed. (D, E) Hemorrhagic bullae on the soles. RND: rheumatoid neutrophilic dermatosis

A 70-year-old Japanese woman presented with a two-week history of a painful rash on her lower extremities. Her medical history included seropositive RA for 14 years, which was successfully treated with a combination of oral prednisolone (PSL) 5 mg daily and tacrolimus hydrate 2 mg daily. However, joint pain and swelling occurred one month before her visit to our department, and she was diagnosed with severe RA. Physical examination revealed multiple painful, tense bullae up to 1.5 cm in diameter, accompanied by pustules, erythematous papules, and erosions on the lower extremities (Figure 1A-1C). A few hemorrhagic bullae were observed on the soles (Figure 1D, 1E). 

**Figure 2:**
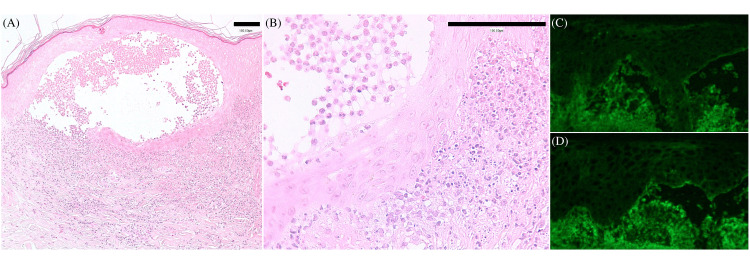
Histological and immunofluorescence findings of bullous RND experienced in our department (A, B) Intraepidermal and subepidermal blister containing numerous neutrophils without acantholytic cells. Marked neutrophilic dermal infiltration showing prominent leukocytoclasis without vasculitis. Bars indicate 100 μm. (C, D) DIF yields negative results for C3 (C), IgG (D), IgA, and IgM. RND: rheumatoid neutrophilic dermatosis; DIF: direct immunofluorescence

A skin biopsy of the blister on the lower leg revealed an intraepidermal and subepidermal blister, containing numerous neutrophils. Marked neutrophilic infiltration, showing prominent leukocytoclasis, was observed in the dermis without vasculitis (Figure 2A, 2B). Direct immunofluorescence (DIF) yielded negative results (Figure 2C, 2D).

Blood tests revealed leukocytosis of 10,460 /μL (normal; 4,000-9,000 /μL) with 65.7% neutrophils and were significant for C-reactive protein (CRP): 2.13 mg/dL (normal; <0.30 mg/dL), rheumatoid factor (RF): 1712 IU/mL (normal; <15 IU/mL), and erythrocyte sedimentation rate: 73 mm/h (normal; <15 mm/h) (Table 1). Neither anti-desmoglein 1, 3 antibodies nor anti-BP180 NC16A antibody was detected.

**Table 1:** Laboratory data of the blood samples on first admission

Parameter	Reference value (female)	On arrival
Aspartate aminotransferase (U/L)	8-35	24
Alanine aminotransferase (U/L)	4-40	18
Sodium (mEq/L)	135–150	138
Potassium (mEq/L)	3.5–5.0	4.9
Urea nitrogen (mg/dL)	7–20	52
Creatinine (mg/dL)	0.40–1.20	1.43
Creatine kinase (U/L)	57–160	96
C-reactive protein (mg/dL)	0–0.30	2.13
White blood cell (/μL)	4,000–9,000	10,460
Neutrophils (%)	45.0-70.0	65.7
Hemoglobin (g/dL)	12.0–15.0	12.5
Platelet (×10^4^ /μL)	15.0–35.0	19.8
Rheumatoid factor (IU/mL)	0-15	1712
Erythrocyte sedimentation rate (mm/h)	0-15	73
Anti-desmoglein 1 antibodies (U/mL)	0-19	<20
Anti-desmoglein 3 antibodies (U/mL)	0-19	<20
Anti-BP180 NC16A antibody (U/mL)	0-8	<9

Bacterial cultures from the blisters were sterile. Therefore, the diagnosis of bullous RND was made. Despite treatment with oral minocycline at 100 mg daily for one week, new skin lesions developed. Treatment was switched to dapsone at 75 mg daily, resulting in the improvement of skin lesions and arthralgia within one week, leaving mild hyperpigmentation. Dapsone was discontinued after three months, and the patient was followed up for one month after cessation, during which no recurrences were observed.

## Discussion

We reviewed English-language articles on bullous and non-bullous RND published between January 1, 1989, and September 1, 2024. A structured literature search of PubMed was performed, using the keywords “(bullous) rheumatoid neutrophilic dermatosis” or “(bullous) rheumatoid neutrophilic dermatitis.” Relevant articles were screened for key terms and included if appropriate. Bullous RND was defined as RND with vesiculobullous lesions, while non-bullous RND was defined as RND without vesiculobullous lesions. 

We identified 40 articles (48 patients) on RND and excluded patients whose iatrogenic RND developed during treatment with interleukin (IL)-6-receptor antagonists [7,8], as drug-induced cases may have distinct clinical or histological features that differ from classic RND. Articles on patients with atypical RND presenting with a solitary skin lesion [9], confined to the same site after trauma [10], and with symmetrically localized erythematous plaques were excluded [11-13]. We also excluded patients with RND who had histological vasculitis [14] and positive DIF results [15], whose diagnoses could not be definitively distinguished from differential diagnoses of RND, including RV.

Ultimately, our study included 10 patients with bullous RND [4,16-23], including an additional patient diagnosed in our department, and 29 patients with non-bullous RND [4,24-46] (Table 2). 

**Table 2:** Summary of the patients with bullous RND (N=10) RND: rheumatoid neutrophilic dermatosis; RA: rheumatoid arthritis; PSL: prednisolone; F: female; M: male; NA: not available.

Author/Year	Age/ Sex	RA duration (years)	Lesion sites	Erythema surrounding the blisters	Effective treatment
Lowe et al., 1992 [4]	56/F	10	Extremities	NA	Naturally healed
Lu et al., 2004 [16]	35/M	18	Extremities	−	Dapsone
Kreuter et al., 2005 [17]	78/F	15	Lower extremities	−	Etanercept
Yamamoto et al., 2010 [18]	65/F	>20	Lower extremities	−	Topical steroid
Fujio et al., 2014 [19]	78/F	2	Extremities and back	−	Etanercept
Soza et al., 2015 [20]	56/F	>10	Extremities	−	Topical steroid
Shin et al., 2015 [21]	64/F	10	Extremities	−	Dapsone
Kosumi et al., 2017 [22]	78/F	2	Lower extremities	Mild	NA
Ha et al., 2021 [23]	45/F	3	Extremities	NA	NA
Our case	70/F	14	Lower extremities	Mild	Dapsone

Tables 3-5 summarize the comparison of RA characteristics (Table 3), clinicopathological features (Table 4), and treatment data (Table 5) between patients with bullous and non-bullous RND. 

**Table 3:** Comparison of the RA characteristics of the patients with bullous and non-bullous RND RND: rheumatoid neutrophilic dermatosis; RA: rheumatoid arthritis. Data are presented as N (%) unless otherwise specified. The mean age at diagnosis of bullous RND was 62.5 years, higher than that of non-bullous RND. Bullous and non-bullous RND were predominantly observed in females. The mean time from RA to bullous RND diagnosis was longer than that of non-bullous RND. The proportion of patients with more than 10 years between the RA and RND diagnoses was 70% in the bullous RND group, which was higher than that in the non-bullous RND group (27%). All patients with RND, except those not described, had severe RA disease activity prior to the development of skin lesions or before consultation. Seropositive RA was noted in all patients with bullous RND, whereas seven patients (24%) with non-bullous RND were seronegative for RA. RND was not complicated by infectious diseases, malignancies, or inflammatory bowel disease and was associated with monoclonal IgA gammopathy and dysglobulinemia, but no hematological malignancies were reported. ^a^ Disease characteristics were compared between subgroups using the Mann-Whitney or Fisher’s exact test as appropriate. Statistical tests were two-sided, and p-values less than 0.05 were considered statistically significant. We used Microsoft^®^ Excel for Mac ver. 16.95.4 (Microsoft Corp., Redmond, WA). ^b^ The number of patients in the bullous and non-bullous RND groups for whom the exact RA duration was known was 8 and 26, respectively. ^c^ The number of patients in the bullous and non-bullous RND groups for whom RA duration could be categorized as either <10 years or ≥10 years was 10 and 26, respectively.

RND types	Bullous RND; N=10	Non-bullous RND; N=29	p-value ^a^
Age, mean (range) years	62.5 (35-78)	49.8 (21-74)	0.031
Sex			0.4
Male	1 (10)	8 (28)	
Female	9 (90)	21 (72)	
RA duration, mean (range) years ^b^	9.3 (2-18)	6.2 (0-20)	0.247
<10 years ^c^	3 (30)	19 (73)	0.026
≥10 years ^c^	7 (70)	7(27)	
RA severity			
High severity	7 (70)	12 (41)	
Low severity or remission	0 (0)	0 (0)	
Not described	3 (30)	17 (59)	
RA characteristics			
Seropositive RA	10 (100)	22 (76)	
Seronegative RA	0 (0)	7 (24)	
Medical history other than RA			
Monoclonal IgA gammopathy	1 (10)	0 (0)	
Dysglobulinemia	0 (0)	1 (3)	
Interstitial pneumonia	1 (10)	0 (0)	
Sjögren’s syndrome	0 (0)	2 (7)	
Others	3 (30)	1 (3)	
None/Not described	5 (50)	25 (86)	

**Table 4:** Comparison of the clinicopathological features of the patients with bullous and non-bullous RND RND: rheumatoid neutrophilic dermatosis All 10 patients with bullous RND had tense vesiculobullous lesions on the lower extremities, six (60%) on the upper extremities, and one (10%) on the back. Although non-bullous RND skin lesions were frequently noted on the lower extremities (76%), as in bullous RND, the trunk was more affected (48%) than in bullous RND. Painful skin lesions were relatively rare in both bullous (20%) and non-bullous RND (28%), and pruritus was similarly uncommon in both bullous (10%) and non-bullous RND (21%). In all patients with RND, the histological features were predominantly neutrophilic dermal infiltrates without leukocytoclastic vasculitis. Leukocytoclasis was observed in more than half of bullous and non-bullous RND patients.

RND types	Bullous RND; N (%), N=10	Non-bullous RND; N (%), N=29
Lesion sites		
Head (neck/face/scalp)	0 (0)	1 (3)
Head+trunk+extremities	0 (0)	3 (10)
Trunk+extremities	1 (10)	10 (34)
Trunk	0 (0)	1 (3)
Extremities	5 (50)	7 (24)
Upper extremities	0 (0)	5 (17)
Lower extremities	4 (40)	2 (7)
Painful rash		
Yes	2 (20)	8 (28)
No (pruritic rash)	1 (10)	6 (21)
No (asymptomatic)	2 (20)	7 (24)
Not described	5 (50)	8 (28)
Papillary dermal edema		
Yes	0 (0)	4 (14)
No	7 (70)	14 (48)
Not described	3 (30)	11 (38)
Leukocytoclasis		
Yes	6 (60)	19 (66)
No	0 (0)	3 (10)
Not described	4 (40)	7 (24)

**Table 5:** Treatment data of the patients with bullous and non-bullous RND RND: rheumatoid neutrophilic dermatosis; PSL: prednisolone; HCQ: hydroxychloroquine; AZM: azithromycin; MTX: methotrexate. Treatment approaches may differ between dermatologists and rheumatologists, as dermatologists primarily focus on managing skin symptoms, whereas rheumatologists concentrate on treating RA itself, considering joint involvement. Therefore, hydroxychloroquine, cyclophosphamide, and etanercept, which are not usually selected by dermatologists, were used for treatment. The total number of patients with spontaneous remission without treatment was 10% in bullous RND, and topical steroids alone were effective in 20% of bullous RND and 7% of non-bullous RND patients. Oral PSL and dapsone were commonly utilized in RND. In addition to oral PSL, combination therapies for RA, including methotrexate and hydroxychloroquine, improved skin lesions in patients with non-bullous RND. The response to treatment can be divided into three patterns: improvement of skin lesions before arthralgia, parallel improvement of both skin lesions and arthralgia (as in our case), or alleviation of arthralgia first.

RND treatment	Bullous RND; N (%), N=10	Non-bullous RND; N (%), N=29
Topical steroids	2 (20)	2 (7)
PSL	0 (0)	4 (14)
Dapsone	3 (30)	4 (14)
Etanercept	2 (20)	0 (0)
Cyclosporine	0 (0)	1 (3)
HCQ	0 (0)	1 (3)
Cyclophosphamide	0 (0)	1 (3)
PSL+MTX	0 (0)	1 (3)
PSL+colchicine+AZM	0 (0)	2 (7)
PSL+MTX+HCQ	0 (0)	1 (3)
Spontaneous remission	1 (10)	0 (0)
Not described	2 (20)	12 (41)

We discovered that the mean age at diagnosis of bullous RND was higher than that of non-bullous RND. This may be due to the longer duration of RA in patients with bullous RND, as 70% of these patients had RA for more than 10 years. Interestingly, we also identified that vesiculobullous lesions were absent in all patients with seronegative RA who developed RND [24-30]. RA can be divided into two types (seropositive and seronegative) based on the presence or absence of RF and anti-citrullinated protein antibodies. Seronegative patients have been reported to demonstrate higher disease activity than seropositive ones [6]. Patients with seronegative RA who developed RND had severe RA before the onset of RND [24-30], and their skin lesions improved with joint involvement [27,28], suggesting a parallel disease course between RND and RA. Therefore, high disease activity in RA may be involved in the onset and exacerbation of RND, even in seronegative patients.

Although the pathophysiology of RND remains unclear, evidence suggests chemokine-mediated neutrophil recruitment (e.g., IL-8 from synovial tissue) plays a key role [37], distinguishing it from SS, where systemic cytokine dysregulation (e.g., G-CSF) is more prominent. Yamamoto et al. reported that serum levels of IL-6 and IL-8 were elevated in a patient with non-bullous RND, but IL-1, IL-2, and tumour necrosis factor-alpha levels were within normal limits [37]. They speculated that IL-8 from synovial tissue, which can attract neutrophils, may contribute to leukocyte accumulation and inflammatory processes in the skin. Kubota et al. reported that immunohistochemistry of specimens from a patient with RND that developed while using an IL-6-receptor antagonist expressed high levels of IL-6 and IL-8, suggesting that the paradoxical drug effect may have been caused by a cytokine imbalance that increased IL-6 [7].

According to a recent literature review of 54 patients, RND presented most frequently as erythematous papules (31%), nodules (15.1%), and/or plaques (13.5%), often distributed in the extremities, and vesiculobullous lesions accounted for 12.7% of patients [3]. In this study, vesiculobullous lesions were found in 25.6% of RND patients. Bullous RND was characterized by tense vesiculobullous lesions without prominent surrounding erythema. Vesiculobullous lesions developed on the extremities, especially on the lower extremities, and no lesions were observed in the chest, abdomen, face, head, and neck. In contrast, skin lesions of non-bullous RND often appeared on the extremities, sometimes on the trunk, and rarely on the head and neck region.

The main differential diagnoses for RND include RV, PG, erythema elevatum diutinum (EED), and SS [3]. RV is a complication of RA that can lead to both systemic symptoms, including fever and peripheral neuropathy, and skin lesions such as ulcers, purpura, hemorrhagic blisters, nodular erythema, and livedo reticularis [5]. The histological findings of RV are leukocytoclastic vasculitis and/or necrotizing granulomatous vasculitis, which can be distinguished from RND. However, one patient with progression from RND to RV has been reported [13]. Since these two conditions can occur simultaneously in an individual, performing skin biopsies, when necessary, and depending on the clinical course, is important. PG is classified as ND and has four clinical variants: ulcerative, pustular, vegetative, and bullous [5,47]. Bullous PG, a rare variant of PG, must be distinguished from bullous RND. Bullous PG is characterized by painful hemorrhagic bullae that develop into rapidly progressive superficial ulcers [47]. Histologically, leukocytoclasis is often absent; however, secondary vasculitis may occur [5]. Bullous RND can be distinguished from bullous PG by a comprehensive assessment, including the lack of rapid progression of tense vesiculobullous lesions to ulcers, and histologically, the presence of leukocytoclasis without vasculitis. Although vesiculobullous formation is rare, EED can present with clinical findings similar to those of RND [4,5,21,38]. However, EED can be distinguished from RND by the presence of vasculitis [4,21,38].

SS is the most challenging differential diagnosis because of its similar clinicopathological presentation to RND. The diagnostic criteria for SS, consisting of two major and four minor criteria, were first proposed by Su and Liu in 1986 [1, 48]. In 1994, von den Driesch published a modification of these diagnostic criteria, requiring both major and two minor criteria for the diagnosis of SS [48]. The major criteria are as follows: (1) abrupt onset of tender or painful erythematous plaques or nodules, occasionally with pustules or blisters, and (2) predominantly neutrophilic dermal infiltrate without leukocytoclastic vasculitis. Minor criteria include the following: (1) preceded by a nonspecific respiratory or gastrointestinal tract infection or vaccination or associated with inflammatory diseases, malignancies, or pregnancy; (2) fever; (3) abnormal laboratory values, including leukocytosis; and (4) excellent response to treatment with PSL or potassium iodide. More recently, Nofal et al. revised the diagnostic criteria for SS and concluded that the abrupt onset of painful erythematous plaques and nodules associated with dense dermal neutrophilic infiltrate was sufficient to confirm the diagnosis of SS [48].

SS and RND share overlapping clinicopathological features, and patients with RND may fulfill the diagnosis of SS. Therefore, based on the characteristics of RND in this study and the differential diagnoses, we propose five diagnostic criteria for RND (Table 6): (1) a definitive diagnosis of RA; (2) high RA disease activity; (3) multiple erythematous papules, nodules, plaques, and/or tense vesiculobullous lesions; (4) predominantly neutrophilic dermal infiltrate without leukocytoclastic vasculitis; and (5) microbial sterility. Since all five of these criteria apply in the reported cases of bullous and non-bullous RND, the possibility of other diseases should be considered if one or more of these criteria are not met. Additionally, treatment response is not included among these criteria, as it varies between individuals, and effective treatments for RND remain poorly understood. We believe that further diagnosis and accumulation of RND cases will lead to a better understanding of treatment and help elucidate its pathophysiological mechanisms.

**Table 6:** The clinicopathological features of bullous and non-bullous RND, and proposed diagnostic criteria and differential diagnosis of RND RND: rheumatoid neutrophilic dermatosis; RA: rheumatoid arthritis. ^a^: Proposed diagnostic criteria for bullous and non-bullous RND. If one or more of these criteria are not met, the possibility of other diseases should be considered.

RND types	Bullous RND	Non-bullous RND
RA duration until onset of RND	≥10 years	<10 years
Clinical features	Tense vesiculobullous lesions without prominent surrounding erythema, predominantly on the lower extremities	Multiple erythematous papules, nodules, and plaques on the trunk and extremities
Histological features	Intraepidermal or subepidermal blister, without acantholysis, and marked neutrophilic infiltration in the dermis without leukocytoclastic vasculitis	Dense neutrophilic dermal infiltrate without leukocytoclastic vasculitis
Diagnostic criteria for RND ^a^	(1) A definitive diagnosis of RA; (2) high RA disease activity; (3) multiple erythematous papules, nodules, plaques, and/or tense vesiculobullous lesions; (4) predominantly neutrophilic dermal infiltrate without leukocytoclastic vasculitis; (5) microbial sterility	
Differential diagnosis	Sweet’s syndrome, rheumatoid vasculitis, pyoderma gangrenosum, erythema elevatum diutinum	

The limitations of this study include the restrictive inclusion criteria for English-language publications, a limited number of cases, and missing data in several studies. The lack of long-term follow-up data highlights the need for prospective studies to assess treatment durability and relapse rates in RND.

## Conclusions

The duration of RA before the onset of bullous RND was often more than 10 years, which was longer than that of non-bullous RND. The clinical characteristics of bullous RND typically include tense vesiculobullous lesions without prominent surrounding erythema, most commonly on the lower extremities. In contrast, even though non-bullous RND lesions were common in the extremities, the trunk was more affected. Since bullous and non-bullous RND have different clinical characteristics, it is important to diagnose and document each subtype of RND to better clarify their clinicopathological differences and guide future treatment strategies.

## References

[1] Nelson CA, Stephen S, Ashchyan HJ, James WD, Micheletti RG, Rosenbach M (2018). Neutrophilic dermatoses: Pathogenesis, Sweet syndrome, neutrophilic eccrine hidradenitis, and Behçet disease. J Am Acad Dermatol.

[2] Ackerman AB (1978). Histologic Diagnosis of Inflammatory Skin Diseases: A Method of Pattern Analysis. https://search.worldcat.org/title/Histologic-diagnosis-of-inflammatory-skin-diseases-:-a-method-of-pattern-analysis/oclc/612298750.

[3] Scotti B, Misciali C, Merli Y, Bardazzi F, Abbenante D, Dika E, Piraccini BM (2024). Rheumatoid neutrophilic dermatitis: a case report and review of the literature. J Eur Acad Dermatol Venereol Clin Pract.

[4] Lowe L, Kornfeld B, Clayman J, Golitz LE (1992). Rheumatoid neutrophilic dermatitis. J Cutan Pathol.

[5] Sayah A, English JC 3rd (2005). Rheumatoid arthritis: a review of the cutaneous manifestations. J Am Acad Dermatol.

[6] Nordberg LB, Lillegraven S, Lie E (2017). Patients with seronegative RA have more inflammatory activity compared with patients with seropositive RA in an inception cohort of DMARD-naïve patients classified according to the 2010 ACR/EULAR criteria. Ann Rheum Dis.

[7] Kubota N, Ito M, Sakauchi M, Kobayashi K (2017). Rheumatoid neutrophilic dermatitis in a patient taking tocilizumab for treatment of rheumatoid arthritis. J Dermatol.

[8] Jost M, Schwarz T, Wehkamp U, Bohne AS, Drerup K (2023). Rheumatoid neutrophilic dermatosis under treatment with the interleukin-6-receptor-antagonist sarilumab in a patient with seropositive rheumatoid arthritis. J Cutan Pathol.

[9] Yoshida Y, Kiryu H, Furue M, Nakayama J, Matsuda T (2003). Rheumatoid neutrophilic dermatitis. J Dermatol.

[10] Żuk G, Jaworecka K, Samotij D, Ostańska E, Reich A (2019). Rheumatoid neutrophilic dermatitis. Reumatologia.

[11] Panopalis P, Stone M, Brassard A, Fitzcharles MA (2004). Rheumatoid neutrophilic dermatitis: rare cutaneous manifestation of rheumatoid arthritis in a patient with palindromic rheumatism. J Rheumatol.

[12] Manriquez J, Giesen L, Puerto CD, Gonzalez S (2016). Rheumatoid arthritis and pseudo-vesicular skin plaques: rheumatoid neutrophilic dermatosis. An Bras Dermatol.

[13] Zhu M, Shen B, Wang P (2024). A case of rheumatoid neutrophilic dermatitis progressed to rheumatoid vasculitis. J Dtsch Dermatol Ges.

[14] Ichikawa MM, Murata Y, Higaki Y, Kawashima M, Furuya T, Saito T (1998). Rheumatoid neutrophilic dermatitis. Eur J Dermatol.

[15] MacAya A, Servitje O, Jucglà A, Peyrí J (2000). Rheumatoid neutrophilic dermatitis associated with pyoderma gangrenosum. Br J Dermatol.

[16] Lu CI, Yang CH, Hong HS (2004). A bullous neutrophilic dermatosis in a patient with severe rheumatoid arthritis and monoclonal IgA gammopathy. J Am Acad Dermatol.

[17] Kreuter A, Rose C, Zillikens D, Altmeyer P (2005). Bullous rheumatoid neutrophilic dermatosis. J Am Acad Dermatol.

[18] Yamamoto T (2010). Coexistence of bullous rheumatoid neutrophilic dermatosis and palmoplantar pustulosis in a patient with rheumatoid arthritis. Int J Dermatol.

[19] Fujio Y, Funakoshi T, Nakayama K, Amagai M, Ohyama M (2014). Rheumatoid neutrophilic dermatosis with tense blister formation: a case report and review of the literature. Australas J Dermatol.

[20] Soza GM, Griffin JR (2015). Acral vesicles and bullae in a patient with severe rheumatoid arthritis. Proc (Bayl Univ Med Cent).

[21] Shin JM, Hong JH, Ko JY, Ro YS, Kim JE (2015). Erythematous vesiculopapular eruptions on the extremities. Clin Exp Dermatol.

[22] Kosumi H, Yanagi T, Shiba K, Sugai T, Nakamura H, Shimizu H (2017). Unusual tense bullae on the legs of a woman with rheumatoid arthritis. Rheumatology (Oxford).

[23] Ha DL, Shin K, Kim HS, Ko HC, Kim BS, Kim MB (2021). A case of vesicobullous rheumatoid neutrophilic dermatosis. J Clin Rheumatol.

[24] Brown TS, Fearneyhough PK, Burruss JB, Callen JP (2001). Rheumatoid neutrophilic dermatitis in a woman with seronegative rheumatoid arthritis. J Am Acad Dermatol.

[25] Yamamoto T, Matsunaga T, Nishioka K (2003). Rheumatoid neutrophilic dermatitis, rheumatoid papules, and rheumatoid nodules in a patient with seronegative rheumatoid arthritis. J Am Acad Dermatol.

[26] Gay-Crosier F, Dayer JM, Chavaz P, Hauser C (2000). Rheumatoid neutrophilic dermatitis/sweet's syndrome in a patient with seronegative rheumatoid arthritis. Dermatology.

[27] Lazarov A, Mor A, Cordoba M, Mekori YA (2002). Rheumatoid neutrophilic dermatitis: an initial dermatological manifestation of seronegative rheumatoid arthritis. J Eur Acad Dermatol Venereol.

[28] Meena L, Bhadu D (2021). Rheumatoid neutrophilic dermatitis as a presenting manifestation of seronegative rheumatoid arthritis. J Clin Diagn Res.

[29] Defaria D, Kroumpouzos G (2004). Rheumatoid neutrophilic dermatitis as presenting sign of seronegative arthritis. Acta Derm Venereol.

[30] IbnIdris Rodwan AA, A Mohammed AG, Adam Essa ME, Abdalla Babker AE, Mohamed Abdelsatir A, Mohammed Elagib E (2022). Neutrophilic dermatoses in a seronegative rheumatoid arthritis patient: A case report. Clin Case Rep.

[31] André R, de Maleissye MF, Costantino F, Sohier P, Clérici T, Hayem G, Breban M (2016). Rheumatoid neutrophilic dermatitis. Joint Bone Spine.

[32] Yali S, Dehui J, Shuhua Z (2012). Rheumatoid neutrophilic dermatitis: a male Chinese case with multiple lesions. Acta Dermatovenerol Croat.

[33] Zhang K, Zhou G, Yu C, Wang K, Zhang F (2016). Pustular rheumatoid neutrophilic dermatitis with Koebner phenomenon. Indian J Dermatol Venereol Leprol.

[34] Kumari I, Dongre A, Mohanty S (2020). Rheumatoid neutrophilic dermatosis: the distinct entity with florid presentation. Indian Dermatol Online J.

[35] Ho CY, Lau TW, Chung HY, Ho CT, Lau CS (2023). A case report of rheumatoid neutrophilic dermatitis in a Chinese woman with seropositive rheumatoid arthritis. Mod Rheumatol Case Rep.

[36] Mashek HA, Pham CT, Helm TN, Klaus M (1997). Rheumatoid neutrophilic dermatitis. Arch Dermatol.

[37] Yamamoto T, Ohkubo H, Nishioka K (1994). Rheumatoid neutrophilic dermatitis. Int J Dermatol.

[38] Scherbenske JM, Benson PM, Lupton GP, Samlaska CP (1989). Rheumatoid neutrophilic dermatitis. Arch Dermatol.

[39] Sánchez JL, Cruz A (1990). Rheumatoid neutrophilic dermatitis. J Am Acad Dermatol.

[40] Hughes JR, Erhardt CC, Clement M (1995). Neutrophilic dermatosis in association with rheumatoid arthritis. Clin Exp Dermatol.

[41] Harkaway K, Elenitas R, Margolis DJ (1995). Erythematous papules in a patient with rheumatoid arthritis. Arch Dermatol.

[42] Hassab-el-Naby HM, Alsaleh QA, Khalifa MA (1996). Rheumatoid neutrophilic dermatitis. Int J Dermatol.

[43] Hirota TK, Keough GC, David-Bajar K, McCollough ML (1997). Rheumatoid neutrophilic dermatitis. Cutis.

[44] Bevin AA, Steger J, Mannino S (2006). Rheumatoid neutrophilic dermatitis. Cutis.

[45] Edgerton CC, Oglesby RJ, Bray D (2006). Rheumatoid neutrophilic dermatitis. J Clin Rheumatol.

[46] Del Priore J, Kassardjian M, Horowitz D (2011). Rheumatoid neutrophilic dermatitis: case report and review. Cosmet Dermatol.

[47] Sakiyama M, Kobayashi T, Nagata Y, Fujimoto N, Satoh T, Tajima S (2012). Bullous pyoderma gangrenosum: a case report and review of the published work. J Dermatol.

[48] Nofal A, Abdelmaksoud A, Amer H (2017). Sweet's syndrome: diagnostic criteria revisited. J Dtsch Dermatol Ges.

